# Laser isotope separation of ^223^Ra

**DOI:** 10.1038/s41598-023-34204-w

**Published:** 2023-04-28

**Authors:** M. V. Suryanarayana

**Affiliations:** Bhabha Atomic Research Centre, Visakhapatnam, Andhra Pradesh India

**Keywords:** Cancer, Atomic and molecular interactions with photons, Electronic structure of atoms and molecules, Theoretical physics

## Abstract

A three-step photoionization has been theoretically studied for the laser isotope separation of ^223^Ra through the following photoionization scheme.

$$\begin{aligned}7s^{2} {}^{1}S_{0} \left( {0.0 \;{\text{cm}}^{ - 1} } \right)\xrightarrow{{714.3185\;{\text{nm}}}} 7s7p\;{}^{3}P_{1}^{o} \left( {13999.3569\;{\text{cm}}^{ - 1} } \right) \xrightarrow{{784.0270\;{\text{nm}}}}\\7s8s\;{}^{3}S_{1} \left( {26754.02 {\text{cm}}^{ - 1} } \right) \xrightarrow{{558/581\;{\text{nm}}}} {\text{Autoionization State}} \to {\text{Ra}}^{ + }\end{aligned}$$

The effect of bandwidth, peak power density of the excitation and ionization lasers, Doppler broadening of the atomic ensemble, number density of the atoms, and charge exchange collisions on the laser isotope separation process has been studied. The optimum system parameters for the separation of ^223^Ra through this photoionization scheme have been derived. The effect of unknown parameters on the degree of enrichment has also been discussed. It has been theoretically shown that it is possible to produce ^223^Ra isotope with 98.5% radio-isotopic purity at a rate of 0.74 μg/h corresponding to the production rate of 435 patient doses per hour. This is the first ever study on the laser isotope separation of Radium isotopes.

## Introduction

Radium was discovered in 1898 by Marie and Pierre Curie. It has more than 20 isotopes and all of them are radioactive. The mass numbers of the known isotopes range from 206 to 230 and their half-lives vary from 182 ns for ^216^Ra to 1600 years for ^226^Ra. Among them, four Radium isotopes ^223^Ra (^235^U decay series), ^224^Ra (^232^Th decay series), ^226^Ra (^238^U decay series), and ^228^Ra (^232^Th decay series) occur in nature as they are members of the radioactive decay series. The Radium isotopes ^223^Ra (T_1/2_ = 11.44 d), ^224^Ra (T_1/2_ = 3.7 d) and ^226^Ra (T_1/2_ = 1600 years) are α-particle emitters, while ^228^Ra (T_1/2_ = 5.75 years) and ^225^Ra (T_1/2_ = 14.9 d) are β-particle emitters.α-particle based radionuclide therapy^1^ using ^223^Ra isotope has been found to be providing survival benefit for castration-resistant prostate cancer (CRPC) patients with symptomatic bone metastases^2,3^. In 2020 alone, 1.4 million new prostate cancer cases and 375 thousand deaths have been reported worldwide^4^. Therefore, survival chances critically dependent on the wider availability of ^223^Ra to the patients.

As mentioned earlier, ^223^Ra occurs naturally only in trace amounts through ^235^U decay; therefore, it is normally produced through irradiation of ^226^Ra in a nuclear reactor^5^. When ^226^Ra isotope is irradiated in a nuclear reactor, it produces ^223^Ra, ^224^Ra, ^227^Ra and ^228^Ra isotopes apart from the ^227^Ac (T_1/2_ = 21.7 years) isotope. The ^227^Ac isotope thus produced decays to ^223^Ra through ^227^Th which is periodically extracted^6^ similar to a moly cow. Due to the long half-life of ^227^Ac, the amount of ^223^Ra produced is rather small. For example, irradiation of one gram of ^226^Ra in a medium flux nuclear reactor (3 × 10^14^ neutrons/cm^2^-sec) for 100 days produces, 14.9 mg of ^227^Ac and 0.11 mg of ^228^Ac (T_1/2_ = 6.13 h). ^228^Ac isotope decays quickly due to its short half-life. After the chemical separation of Ac and cooling for 100 days, the ^227^Ac produces 20 μg of ^223^Ra. With no carrier added specific activity of ^223^Ra being 1893.7 TBq/gm; a typical dose requirement of 55 kBq/kg of body weight, given at 4 week intervals of 6 doses^7,8^; each dose for a 60 kg patient corresponds to 1.7 ng of ^223^Ra. Therefore, about 12,000 doses can be produced every 100 days using the cow. Though, this is an easy and efficient method for the production of ^223^Ra, the valuable ^223^Ra produced during the 100 days of irradiation of ^226^Ra is not utilized. For example, when one gram of ^226^Ra is irradiated in a medium flux reactor for 100 days; it produces 16 μg of ^223^Ra corresponding to ~ 9400 doses. Due to the large scale requirement of the ^223^Ra isotope, it is perhaps pertinent to recover ^223^Ra isotope from the irradiated ^226^Ra isotope mixture.

Among the co-produced isotopes during 100 days irradiation in a medium flux reactor, namely ^223^Ra, ^224^Ra, ^227^Ra and ^228^Ra; the ^227^Ra isotope decays quickly due to its short half-life (T_1/2_ = 47 m). Thus, the residual Radium isotope mixture consists of ^223^Ra (1.66 × 10^−3^%), ^224^Ra (1.01 × 10^−2^%), ^226^Ra (99.999%) and ^228^Ra (3.96 × 10^−6^%). However, in order to utilize this ^223^Ra for radionuclide therapy, it needs to be separated from rest of the isotopes of Radium.

In present work, the possibility of separation of ^223^Ra from the remaining Radium isotopes through Atomic Vapor Laser Isotope Separation (AVLIS) method has been examined. The density matrix formalism has been adopted for the calculation of ionization efficiency of the isotopes under various conditions from which the degree of enrichment has been calculated. The optimum conditions for the efficient separation of ^223^Ra have been derived. This is the first ever study reported on the laser isotope separation of ^223^Ra. All the wavelengths mentioned in the article are vacuum wavelengths.

### AVLIS of radium

An excellent compilation of spectroscopic data of Radium can be found in Ref.^9^. Radium belongs to the alkaline earth metal group having a ground state electronic configuration of [Rn]7s^2 1^S_0_. Radium has ionization energy of 42,573.36 cm^−1^ (5.3 eV). Since energy of a photon in the visible region is about 2 eV, it requires a three-step laser photoionization process for the selective ionization of Ra. Most of the known transitions originating from the 7s^2 1^S_0_ are in the UV wavelength region except for the $7{s}^{2}{}^{1}{S}_{0} (0.0\, {\mathrm{cm}}^{-1}) \stackrel{482.7277\, \mathrm{nm}}{\to } 7s7p {}^{1}{P}_{1}^{o}\left(20715.6142\, {\mathrm{cm}}^{-1}\right)$ and $7{s}^{2}{}^{1}{S}_{0} (0.0\, {\mathrm{cm}}^{-1}) \stackrel{714.3185 \,\mathrm{nm}}{\to } 7s7p {}^{3}{P}_{1}^{o}\left(13999.3569\, {\mathrm{cm}}^{-1}\right)$ transitions. Isotope shifts of both the transitions are similar in magnitude^10^. Sebastian Raeder et al.^11^ have used 482.7277 nm transition for the photoionization of Ra which requires a frequency doubled Ti:Sapphire lasers for the excitation and photoionization process. Since frequency doubling is not an efficient process, such photoionization scheme cannot be used for AVLIS process. Day Goodacre et al.^12^ have used the following three step photoionization scheme wherein two Ti:Sapphire lasers have been used for the excitation and the dye laser has been used for the ionization.

#### Photoionization scheme

$$7{s}^{2}{}^{1}{S}_{0} (0.0\,{\mathrm{cm}}^{-1}) \xrightarrow{{714.3185\,{\text{nm}}}} 7s7p {}^{3}{P}_{1}^{o}\left(13999.3569\, {\mathrm{cm}}^{-1}\right)\xrightarrow{{784.0270\,{\text{nm}}}}$$

$$7s8s {{}^{3}S}_{1} \left(26754.02\,{ \mathrm{cm}}^{-1}\right) \xrightarrow{{558/581\,{\text{nm}}}} \mathrm{Autoionization\,State }\stackrel{}{\to }{\mathrm{Ra}}^{+}$$

Lynch et al.^13^ have measured isotope shifts for the 714.3185 nm transition. The isotope shifts relative to the ^223^Ra isotope were reported to be ^224^Ra (− 2706 MHz), ^226^Ra (− 8798 MHz) and ^228^Ra (− 14,719 MHz). The isotope shifts for the 784.0270 nm transition have not been reported so far, therefore, the isotope shift has been considered as zero. The effect of magnitude of isotope shifts of second excitation transition on the laser isotope process will be discussed in the subsequent sections. The atomic hyperfine structure constants of ^223^Ra for the resonant energy levels of the photoionization scheme are tabulated in Table 1.

**Table 1:** A table of hyperfine structure constants of ^223^Ra isotope energy levels.

Level	^223^Ra		Reference
	A (MHz)	B (MHz)	
7s7p ^3^P°_1_ (13,999.3569 cm^−1^)	1202.5	− 472.1	^13^
	1206.6	− 467.63	^14^
7s8s ^3^S_1_ (26,160.293 cm^−1^)	1504.3	0.0	^14^

The hyperfine pathways of the two-step resonance three-photon ionization of ^223^Ra isotope are tabulated in Table 2. Among them, $F = \frac{3}{2} \to F^{^{\prime}} = \frac{5}{2} \to F^{^{\prime\prime}} = \frac{5}{2}$ (where F, Fʹ and Fʺ correspond to the F-quantum numbers of the ground, first and second excited states respectively) hyperfine pathway corresponds to the most intense hyperfine pathway corresponding to the resonance frequency of (1685.7 MHz, 570.7 MHz). Therefore, in the present work, selective photoionization of ^223^Ra has been investigated through to this hyperfine pathway.

**Table 2:** A table of hyperfine pathways of ^223^Ra isotope.

S. no.	714.3185 nm transition*				784.0270 nm transition*			
	Fʹ	Fʺ	Freq. (MHz)	Relative Intensity	Fʺ	Fˈˈˈ	Freq. (MHz)	Relative Intensity
1	$\frac{3}{2}$	$\frac{1}{2}$	− 3596.4	33.3	$\frac{1}{2}$	$\frac{1}{2}$	− 164.4	7.9
2	$\frac{3}{2}$	$\frac{1}{2}$	− 3596.4	33.3	$\frac{1}{2}$	$\frac{3}{2}$	2092.1	39.7
3	$\frac{3}{2}$	$\frac{3}{2}$	− 730.4	66.6	$\frac{3}{2}$	$\frac{1}{2}$	− 3030.4	39.7
4	$\frac{3}{2}$	$\frac{3}{2}$	− 730.4	66.6	$\frac{3}{2}$	$\frac{3}{2}$	− 773.9	12.7
5	$\frac{3}{2}$	$\frac{3}{2}$	− 730.4	66.6	$\frac{3}{2}$	$\frac{5}{2}$	2986.9	42.8
6	$\frac{3}{2}$	$\frac{5}{2}$	1685.7	100.0	$\frac{5}{2}$	$\frac{3}{2}$	− 3190.0	42.8
7	$\frac{3}{2}$	$\frac{5}{2}$	1685.7	100.0	$\frac{5}{2}$	$\frac{5}{2}$	570.7	100.0

*Fʹ, Fʺ and Fˈˈˈ are the hyperfine levels of the first, second and third excitation states.

**Figure 1:**
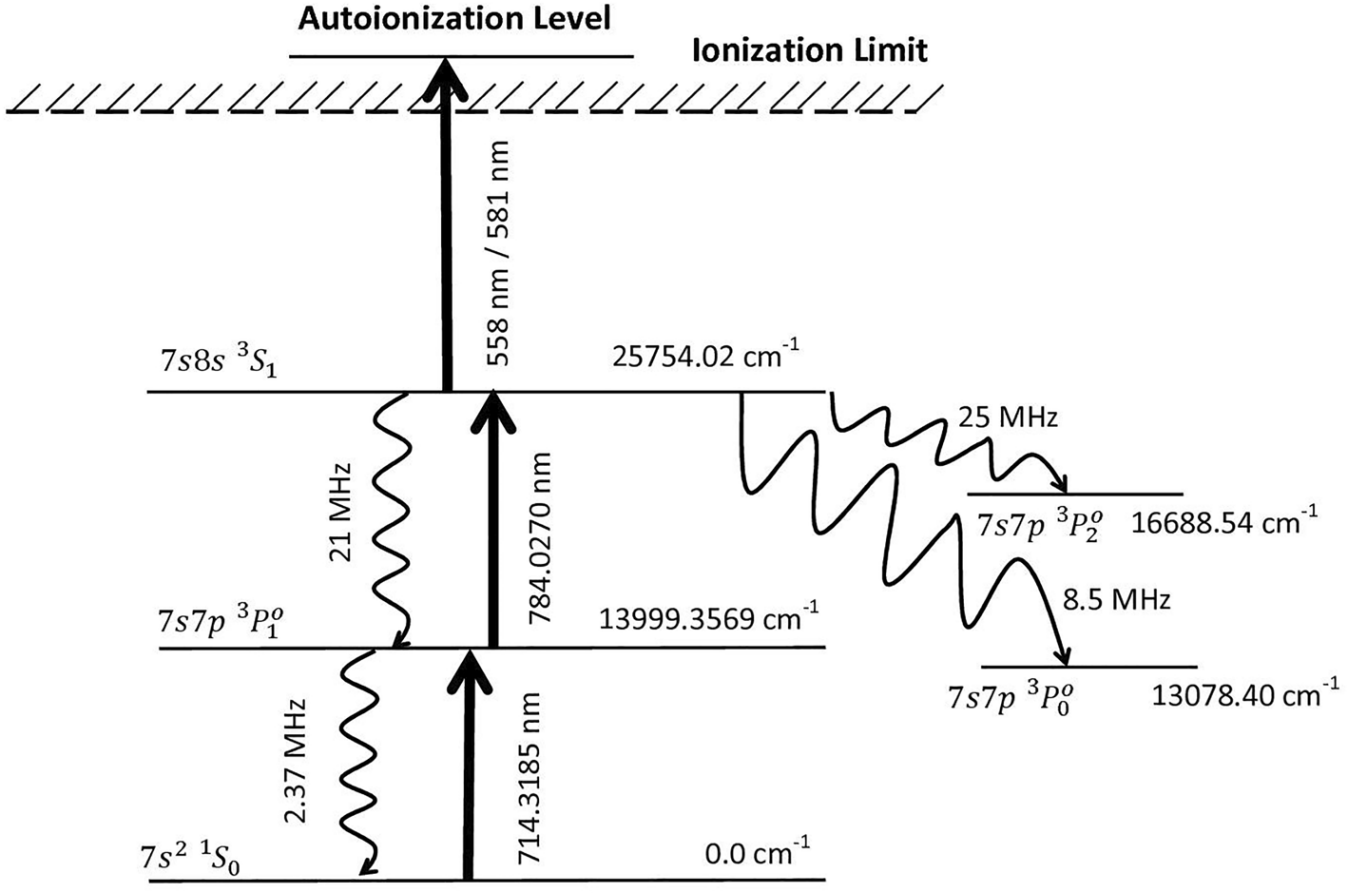
Schematic diagram of the photoionization scheme of Radium (not to scale).

The schematic of the photoionization scheme has been shown in Fig. 1. The atoms from the 7s^2 1^S_0_ (0.0 cm^−1^) ground state are excited into the 7s7p ^3^P°_1_ (13,999.3569 cm^−1^) state using a pulsed (pulse width = 50 ns) Ti:Sapphire laser having a pulsed repetition frequency of 10 kHz which is tuned to the 714.3185 nm transition. The atoms from the 7s7p ^3^P°_1_ (13,999.3569 cm^−1^) are further excited into 7s8s ^3^S_1_ (25,754.02 cm^−1^) using a similar Ti:Sapphire laser tuned to the 784.0270 nm transition and eventually ionized through an autoionization transition. The decay rates to the resonant lower levels and trapped levels are also shown in Fig. 1. For the present work, the excitation lasers have been considered to be unpolarized.

Density matrix equations accurately describe laser-atom interactions in a multi-step laser photoionization process^15^. The population dynamics in a three-step ladder excitation of an atom described by the coupled density matrix equations are published in Ref.^16^.

The excitation into autoionization state is considered as incoherent process, which is induced by ionizing laser at a rate ${\gamma }_{i}= \sigma \Phi$, where σ is the photoionization cross-section and ϕ is the flux of the ionization laser. For a typical auto-ionization cross-section of 1 × 10^−16^ cm^2^, the ionization rate γ_i_ is calculated to be 1.46 × 10^3^ *I* Sec^−1^, where I is the power density of the ionization laser in W/cm^2^.

According to the phase diffusion model, the laser spectrum is Lorentzian near the centre with full width at half maximum (FWHM) γ_ι_ and has a cut-off around β_ι_. For the detunings $\Delta\upiota \ll \mathrm{\beta \iota },$ these terms reduce γ_ι_; while $\Delta\upiota \gg \mathrm{\beta \iota },$ the laser appears to be monochromatic.

The effect of the laser bandwidth and its lineshape are included in the terms^17,18^

1 $$2.{\gamma }_{i}\frac{{\beta }_{i}^{2}}{{\Delta }_{i}^{2}+{\beta }_{i}^{2}},\mathrm{i}=\mathrm{1,2}$$

For a typical evaporation temperature of 1173° C for Ra, the most probable velocity of ^226^Ra is 326.1 m/sec. At this temperature, the vapor pressure of Radium is 100 mbar. When the full angle divergence of the atomic ensemble of Radium is limited to 30°, the number density of the atoms will be ~ 1 × 10^12^ atoms/cm^3^ at 100 mm above the exit orifice of the atom source. In this region, the atomic beam diameter in the perpendicular plane of the laser propagation axis will be 55 mm. Under these conditions, the flux averaged irradiation probability of the atomic beam is 0.99.

For the entire calculations in this work, the laser has been considered to be having a Gaussian pulse-width of 50 ns, with no delay between the pulses and all the lasers are considered to be co-propagating. Initially, the population of the ground state has been set to one. The coupled density matrix equations are then integrated using the standard numerical integration method for the set conditions for the entire duration of the laser–atom interaction. At the end of the laser interaction, the population of the ion state corresponds to the ionization efficiency of the photoionization process. For the inclusion of Doppler broadening of the atomic ensemble in the calculations, divergence angle of the atomic ensemble has been segmented into a minimum of 30 angular groups; and each angular group has been segmented into a minimum of 30 velocity groups. The frequency scale in this article is referenced to the centre of gravity of the ^223^Ra transition unless stated otherwise.

## Results and discussion

In an atomic vapor laser isotope separation process, the primary objective is to obtain high isotope selectivity (resulting in high degree of enrichment) and high ionization efficiency (resulting in high production rate). Further, the above objective shall be achieved with the simplest possible system configuration. Unfortunately, many a times, achieving these objectives requires mutually excluding conditions. Therefore, one needs to optimize the system parameters such as bandwidth and power of the excitation lasers, Doppler broadening of the atomic ensemble so that high degree of enrichment can be achieved without significant compromise in production rates. The optimization of the system parameters has been carried out as described below.

**Figure 2:**
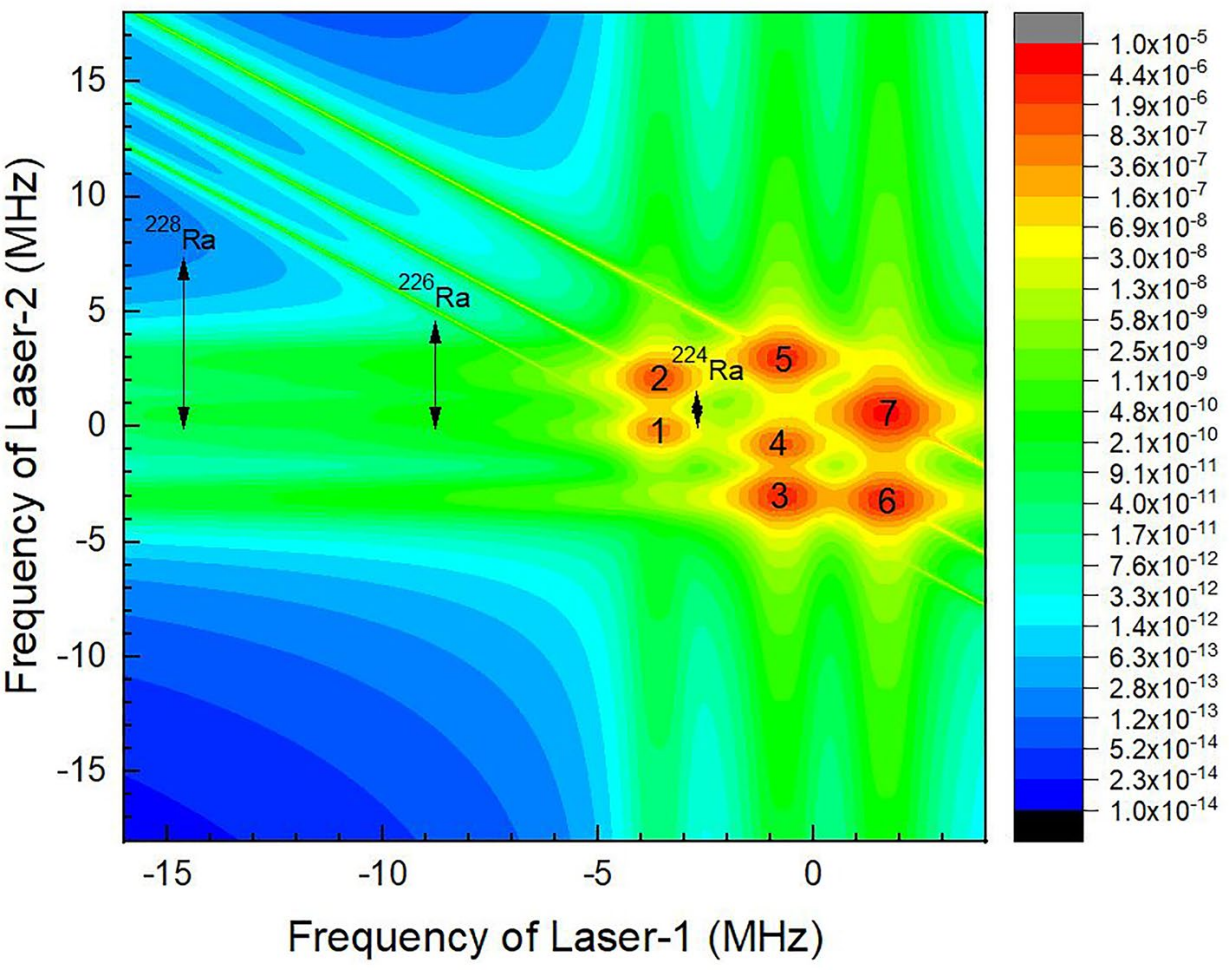
Two dimensional contour of the ionization efficiency of ^223^Ra isotope under Doppler free excitation. The bandwidth of excitation lasers is 500 MHz, peak power density of excitation lasers is 10 W/cm^2^. The resonance frequency positions of the hyperfine pathways were numbered according to Table 2. The range of resonance freqency position of ^224^Ra, ^226^Ra, and ^228^Ra isotopes for the second excitation transition is also shown.

First, the dependence of ionization efficiency on the frequency of excitation lasers has been studied. The frequency of both the excitation lasers has been varied by several GHz from it resonance frequency and the resultant ionization efficiency data has been plotted as the two-dimensional contour plot. A typical plot obtained for the ^223^Ra isotope has been plotted in Fig. 2. The hyperfine pathways were numbered as per serial numbers in Table 2. All the hyperfine pathways were found to be at the expected resonance frequency positions. The horizontal and vertical ridges in the contour correspond to the wing overlap excitations of the first and second excitation transitions, respectively, while, the diagonal ridges correspond to the coherent two-photon ionization. As mentioned earlier, the isotope shifts for the second excitation transition have not been reported so far. The possible resonance frequency position range of even ^224^Ra, ^226^Ra and ^228^Ra isotopes for the second excitation transition have been marked in Fig. 2. A detailed discussion on the two-dimensional contours can be found in Ref.^16,19^. When the lasers are tuned to the $F = \frac{3}{2} \to F^{^{\prime}} = \frac{5}{2} \to F^{^{\prime\prime}} = \frac{5}{2}$ hyperfine pathway (marked as 7 in Fig. 2), the ionization efficiency has been found to be 1.6 × 10^−5^. The low ionization efficiency of ^223^Ra is due to the low peak power density of the excitation and ionization lasers (10 W/cm^2^). From the transition probability values of 2.37 × 10^6^ Sec^−1^ and 21 × 10^6^ Sec^−1^ for the first and second excitation transitions; the saturation power density of the transitions was calculated to be 0.41 mW/cm^2^ and 2.7 mW/cm^2^ respectively. Though the peak power density (10 W/cm^2^) used was much higher than the saturation power density of the excitation transitions, the ionization efficiency is not adequate enough for the efficient laser isotope separation process. Therefore powers of both excitation and ionization lasers must be increased to enhance the ionization efficiency. When the powers of the excitation lasers are increased beyond the saturation power, saturation broadening occurs. The saturation broadening (Γ_sat_) can be calculated using the expression

2 $${\Gamma }_{\mathrm{sat}}= {\Gamma }_{0 }\sqrt{1+\frac{{\mathrm{I}}_{\mathrm{laser}}}{{\mathrm{I}}_{\mathrm{sat}}}}$$

When the powers of the excitation lasers are 10 W/cm^2^; saturation broadening for the excitation transitions was calculated to be 372 MHz, 1274 MHz respectively. Since saturation broadening is much smaller in magnitude than the isotope shift of the non-target isotopes, the powers of the excitation lasers can be increased without significant loss in the isotope selectivity. When the powers of excitation lasers are increased to very high values, the isotope selectivity is lost significantly due to increased saturation broadening resulting in increased ionization of non-target isotopes. Therefore, at certain peak power density values of the excitation and ionization lasers, isotope selectivity and ionization efficiency reach their optimum values. In order to find such optimum values, a series of calculations of ionization efficiency has been carried out for all the constituent isotopes for different lasers bandwidths and varying peak power density of all the three lasers (Table 3). The degree of enrichment of the target isotope has been calculated using the expression

3 $$\mathrm{Degree\,of\,enrichment }\,\left(\mathrm{\%}\right)\,\mathrm{ of }{}^{223}\mathrm{Ra }=\left\{\frac{{\upeta }_{223}{*\mathrm{A}}_{223}}{\sum_{i}^{all}{\upeta }_{\mathrm{i}}{*\mathrm{A}}_{\mathrm{i}}}\right\}*100$$

where η corresponds to the ionization efficiency of the isotope, A is the fractional abundance and *i* is the isotope mass index.

**Table 3:** A table of the degree of enrichment of ^223^Ra for various bandwidths of the excitation lasers under Doppler free condition. Peak power density of the excitation and ionization lasers is 10, 10, 10,000 W/cm^2^.

Bandwidth (MHz) of the excitation lasers	Ionization efficiency of ^223^Ra (${\upeta }_{{223}_{\mathrm{Ra}}}$) × 10^–2^	Degree of enrichment (%) ^223^Ra
50	1.83	93.3
100	1.58	81.3
250	0.99	54.4
500	0.53	20.7

For peak power density optimization of excitation and ionization lasers, the peak power density of excitation lasers has been sequentially varied between 10 and 60 W/cm^2^ in steps of 10W/cm^2^; while the peak power density of the ionization laser varied between 1 kW and 10 kW/cm^2^ in steps of 1 kW/cm^2^. For these calculations, bandwidth of the excitation lasers was set to 500 MHz. Highest ionization efficiency has been obtained for 60, 60, 10,000 W/cm^2^ (ionization efficiency = 1.77 × 10^−2^), however, in this case, the degree of enrichment was found to be 9.2%. On the other hand, when the powers of the lasers set to 10, 10, 1000 W/cm^2^ (lowest values in this calculation region) the ionization efficiency was found to be 5.3 × 10^−4^ while the degree of enrichment was 20.7%. In AVLIS, it is always a compromise between the ionization efficiency and degree of enrichment. At the powers of 10, 10, 10,000 W/cm^2^; the ionization efficiency if found to be 5.3 × 10^−3^ (one order higher than the lowest value) and the degree of enrichment remained at 20.7%. Therefore, these values are considered to be optimum.

A series of calculations having been carried out varying the bandwidth of the excitation lasers under the optimum peak power conditions (Table 3). From Table 3, it can observed that when narrowband lasers (bandwidth = 50 MHz) are employed for the isotope separation process, the degree of enrichment of the target isotope reaches a value of > 90%. With increase in the bandwidth, the degree of enrichment degrades. When the bandwidth of the excitation laser is set to 500 MHz, the degree of enrichment was found to be 20.7%. It can also be observed that the ionization efficiency of the target isotope also falls by ~ 70% with increase in the laser bandwidth. Since these calculations have been carried out under the Doppler free conditions, these should be considered as upper limits. The effect of Doppler broadening on the degree of enrichment and ionization efficiency has been dealt as described below.

Atomic transitions of an atomic ensemble exhibit Doppler broadening. Doppler broadening arises due to the velocity and angular distributions of atoms exiting the atom source. The flux-velocity distribution of atomic ensemble is given by the following expression

4 $$\phi \left(v\right)=2\left(\frac{{v}^{3}}{{v}_{mp}^{4}}\right).{e}^{-\left(\frac{{v}^{2}}{{v}_{mp}^{2}}\right)}dv$$

where v_mp_ is the most probable velocity. At 4 × v_mp_, the relative flux drops to the value of ~ 10^−7^ of the maximum.

The divergence of the atomic beam can be calculated using the collimation ratio of the *“canal” type* atom source.

5 $$Collimation\,ratio= \frac{l}{d}$$

where “*l*” is the length and “*d*” is the internal diameter of the atom source.

The collimation ratio can be further increased by incorporating the additional aperture in the plane perpendicular to the atom propagation axis; however, this could result in the loss of throughput of atoms. Therefore, atom sources can be built with the requisite full angular divergence

6 $$\theta =2{\times Tan}^{-1} \left(\frac{d}{l}\right)$$

When Radium is evaporated at the temperature of 1173° C, the Doppler broadening of the first and second excitation transitions for the freely expanding atoms will be 761 MHz and 693 MHz respectively. When the full angular divergence is limited to 30°, the residual Doppler broadening of the transitions falls to 376 MHz and 343 MHz respectively. The Doppler broadening of the transitions affects the AVLIS process in two ways. First, when the Doppler broadening of the atomic ensemble is larger than the bandwidth of the excitation lasers; the larger velocity groups which are having larger Doppler shifted resonances lie far away from the rest frame resonance frequency, therefore they are unlikely to be excited and ionized by lasers. As a result, production rates are affected. On the other hand, when the bandwidth of the excitation lasers is larger than the Doppler broadening of the atomic ensemble; the photon frequencies at the pedestal of the laser bandwidth are not in resonance with atoms. As a result photon economy is lost. Therefore, in order to find an optimum values for the bandwidth of the excitation lasers and Doppler broadening, a series of calculations of ionization efficiency and degree of enrichment have been carried out varying the angular divergence of the atomic ensemble, bandwidth and peak power density of the excitation lasers. In order to incorporate Doppler broadening into the calculations, the flux-velocity distribution of the atomic ensemble has been calculated according to the Eq. (4). Further, the entire atomic ensemble has been segmented in to a minimum of 30 velocity groups and each velocity group has been segmented into 30 angular groups. The results are tabulated in Table 4. From Table 4, as expected, the degree of enrichment falls with increase either in the bandwidth of the excitation lasers or in the angular divergence of the atomic ensemble. In case of broadband lasers (bandwidth = 500 MHz), the effect of angular divergence on the degree of enrichment and ionization efficiency is not significant. In case of relatively narrowband lasers (≤ 250 MHz), the loss in the degree of enrichment and ionization efficiency has been found to be up to 19% (100 MHz case) and 52% (50 MHz case) respectively. When the bandwidth of the excitation lasers has been increased from 50 to 500 MHz, the degree of enrichment has degraded from ~ 85 to ~ 20%. On the whole, the full angle divergence value of 30° is considered as optimum for the selective ionization of ^223^Ra.

**Table 4:** Table of variation in the degree of enrichment of ^223^Ra with the increase in the angular divergence of the atomic beam. Peak power density of the excitation and ionization lasers is 10, 10, 10,000 W/cm^2^ respectively.

Bandwidth of excitation laser (MHz)	Full angle divergence (degrees)	Ionization efficiency of ^223^Ra (${\upeta }_{{223}_{\mathrm{Ra}}}$) × 10^–2^	Degree of enrichment of ^223^Ra (%)
50	10°	1.80	84.7
	30°	1.50	81.2
	60°	0.94	68.6
100	10°	1.56	81.0
	30°	1.26	76.1
	60°	0.82	61.9
250	10°	0.99	54.2
	30°	0.92	51.1
	60°	0.69	42.0
500	10°	0.53	20.7
	30°	0.53	20.7
	60°	0.48	19.9

After obtaining the optimum values for the peak power densities of the excitation and ionization lasers and full angle divergence value of the atomic beam, the effect of charge exchange collisions has been studied. Charge exchange collisions play a vital role in AVLIS process. The photoions formed during the laser-atom interaction process are extracted using an electric field gradient. During extraction, when the atoms traverse through the atomic ensemble, they undergo charge exchange collisions with unionized atomic vapor. Probability of charge exchange can be calculated using the expression

7 $$Charge\,exchange\,probability\,p=1-{e}^{-\sigma *dist*den}$$

where σ is the resonant charge exchange cross-section (cm^2^), dist is the distance traversed by the photoions prior to the collection at the collector (cm) and den is the number density of the atoms (atoms/cm^3^).

Resonant charge exchange cross-section can be calculated using the following formula^20^

8 $$\sigma \left(v\right)=\left(1.81\times {10}^{-14}-2.12\times {10}^{-15}{.log}_{10}v\right).{\left(\frac{IP}{13.6}\right)}^{-1.5}$$

where v is the velocity of the ion in cm/sec and IP is the ionization potential of the element in eV. From the ionization potential of Ra (5.2789 eV) and the most probable velocity (328.29 m/sec), the resonant charge exchange cross–section value can be calculated to be 3 × 10^−14^ cm^2^. Therefore for the present computations the resonant charge exchange cross–section value has been taken as 1 × 10^−14^ cm^2^. For this resonant charge exchange cross section, for a number density of 1 × 10^12^ atoms/cm^3^ and a 50 mm extraction region width, the probability of charge exchange collisions is calculated to be 5%.

**Figure 3:**
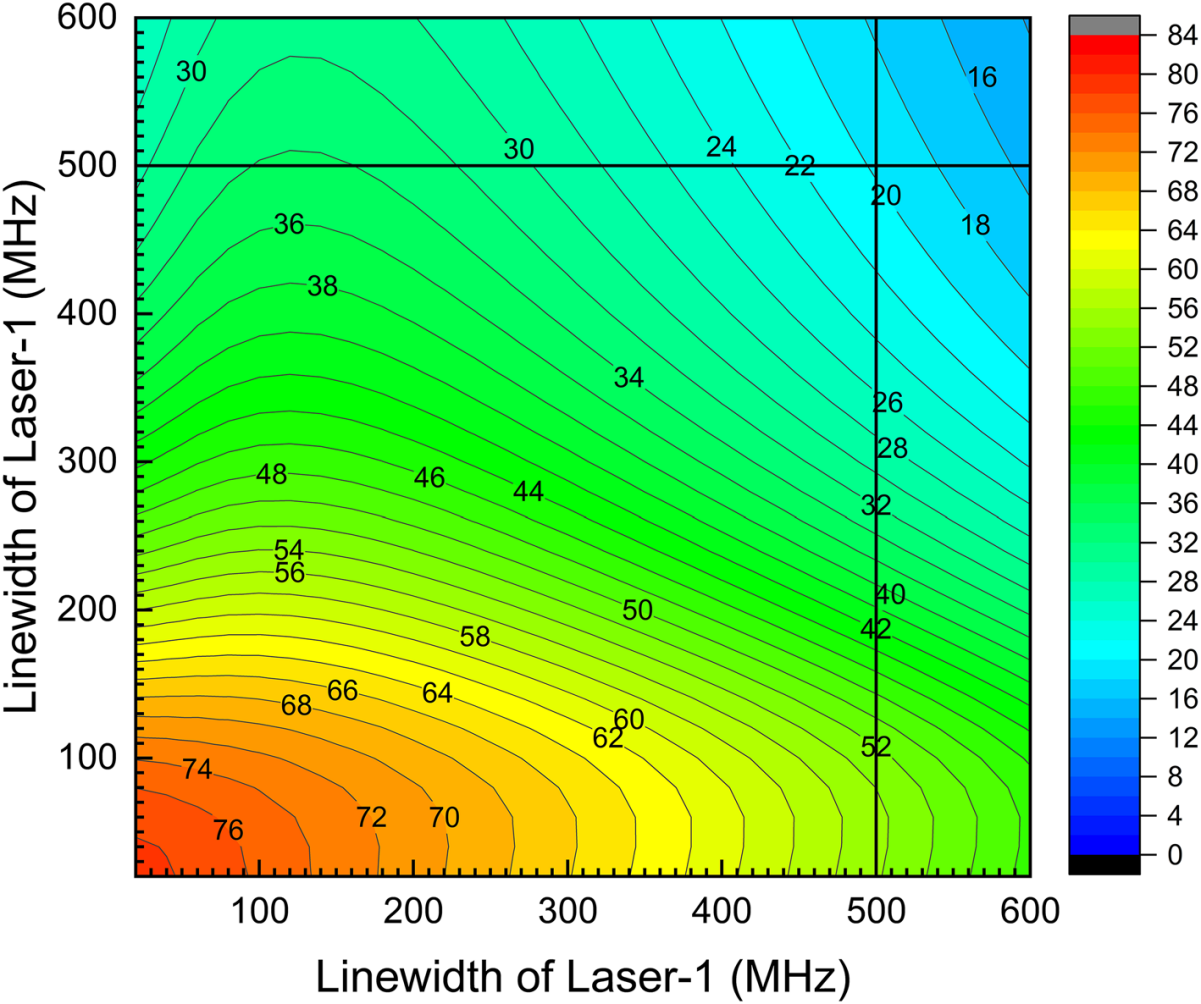
A plot of degree of enrichment of ^223^Ra isotope with variation in the bandwidth of excitation lasers. Full angle divergence of the atomic beam is 30°. Peak power densities of the first, second and third excitation lasers are 10, 10, 10,000 W/cm^2^ respectively. Atomic number density is 1 × 10^12^ atoms/cm^3^.

A series of calculations have been carried out varying the bandwidth of the excitation lasers for different atomic number densities ranging from 10^11^ to 10^13^ atoms/cm^3^ and the degree of enrichment has been calculated. It has been found that up to the number densities of 1 × 10^12^ atoms/cm^3^ the degree of enrichment remained somewhat unaffected. A typical two-dimensional contour of the degree of enrichment with variation in bandwidth of the excitation lasers has been shown in Fig. 3 for the number density of 1 × 10^12^ atoms/cm^3^. The computed degree of enrichment for the 500 MHz bandwidth was found to be 19.7% for ^223^Ra. Therefore, the number density of 1 × 10^12^ atoms/cm^3^ has been considered as an optimum value for the enrichment of ^223^Ra.

Production rates can be calculated using the following expression

9 $$P \left(\frac{g}{hour}\right)=2.827\times {10}^{3}\times \left({dia}^{2}.p.len.den.A.f.\eta .i.n.\frac{M}{{N}_{A}}.PRF\right)$$

where dia is the laser beam diameter (cm), *p* fractional population of the ground level, len is the length of the laser-atom interaction region (cm), den is the number density of atoms in the interaction region (atoms/cm^3^), A is the fractional abundance of the target isotope, f is the fractional flux (flux relative to the flux of unhindered atomic beam), η is the ionization efficiency (derived from the density matrix calculations), *i* is the irradiation probability, n is the number of passes of the laser beam through the atomic ensemble, M is the atomic mass of the target isotope (grams), N_A_ is the Avogadro number (6.02214076 × 10^23^) and PRF is the pulse repetition frequency of the lasers (Hz).

For the values of dia = 5.5 cm, p = 1, len = 27 cm, den = 1 × 10^12^ atoms/cm^3^, A = 1.66 × 10^−3^, f = 1, η = 5.3 × 10^−3^, i = 0.99, n = 1, M = 223, PRF = 10 kHz, the production rate is calculated to be 0.74 μg/h. This corresponds to the production rate of 435 patient doses per hour.

### Effect of unknown parameters on the laser isotope process

As mentioned in the previous section, isotope shift for the second excitation 784.0270 nm transition has not been measured so far. The isotope shift of this transition may have significant influence on the degree of enrichment of the laser isotope separation process. Therefore this aspect has been studied further. The isotope shift for the ^1^S_0_-^3^P°_1_ and ^3^P°_1_-^3^S_1_ transitions of the isotopes of iso-electronic elements viz. Ca^21,22^ and Sr^23^ have been analyzed through King’s plot analysis^24^. The isotope shift of transition comprises of field and mass shifts which can be expressed as^23^

10 $${\delta \gamma }^{{A}^{^{\prime}},A}\approx F. {\delta \langle {r}^{2}\rangle }^{{A}^{^{\prime}},A}+M.\left(\frac{{A}^{^{\prime}}- A}{{A}^{^{\prime}}.A}\right)$$

where F, M are the field shift and mass shift parameters respectively and ${\delta \langle {r}^{2}\rangle }^{{A}^{^{\prime}},A}$ is the difference in the mean square nuclear charge radii.

The King’s plot analysis of the ^1^S_0_-^3^P°_1_ and ^3^P°_1_-^3^S_1_ transitions of iso-electronic elements like Ca and Sr has shown that the field shift of the second excitation transition is about 50–60% of the first excitation transition. Therefore, the same can be expected in case of Ra transitions.

Further, unlike Ca and Sr transitions, Ra transitions are solely dominated by field shift. For example, Lynch et al.^13^ have reported the field shift parameter of F = − 28.75 GHz/fm^2^ and mass shift parameter M = − 305 GHz.u for the 714.3185 nm transition of Ra. From the known values of mean square nucleus charge radii of Radium isotopes, field and mass shifts have been calculated. The mass shift has been found to be about ~ 0.2% of the field shift value; hence, mass shift contribution can be ignored. Therefore, the isotope shift of the second excitation transition has been taken as 50% of the first excitation transition.

**Figure 4:**
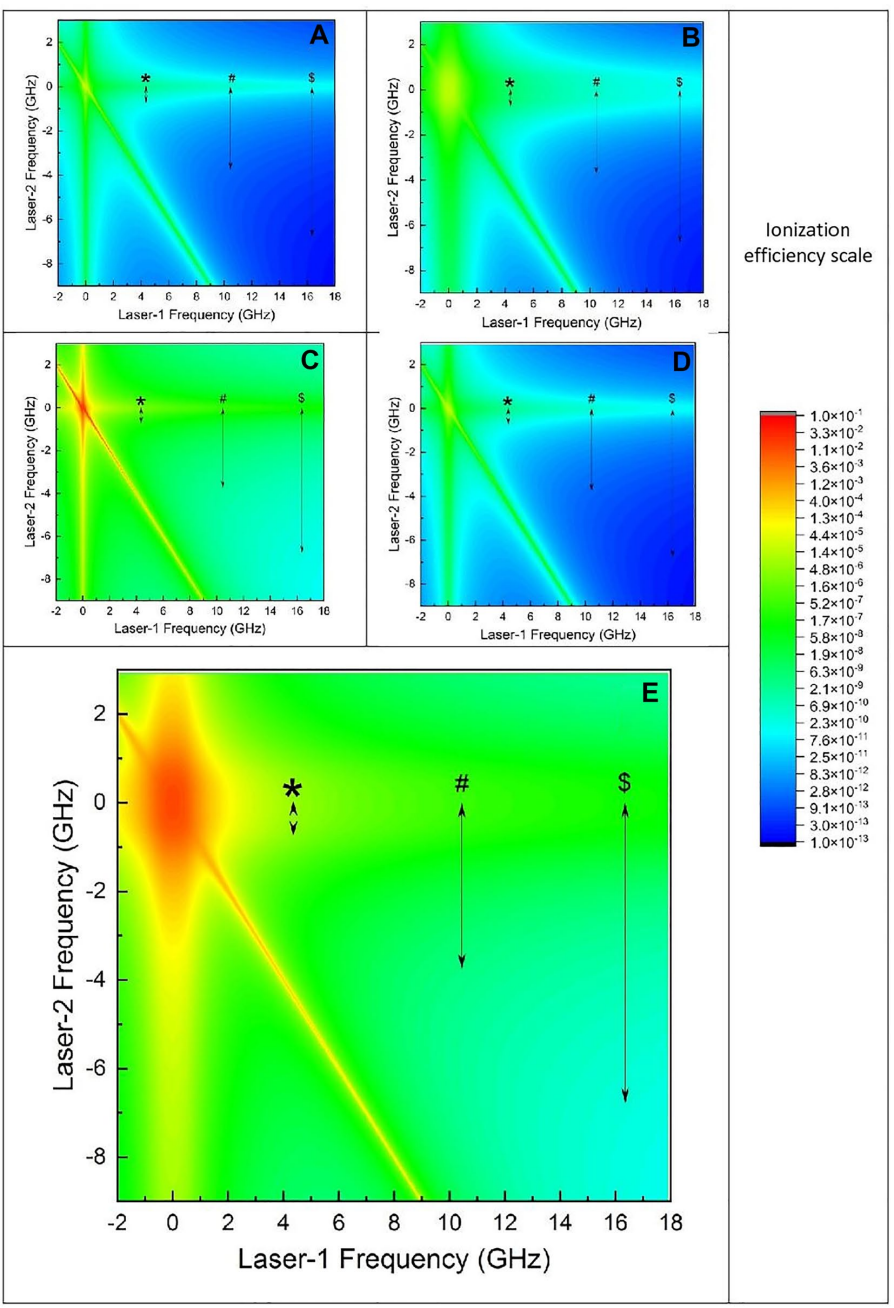
Two dimensional contour of ionization efficiency of even isotope of Radium. The frequency scale is referenced to the resonance frequency of the even isotope. The resonance position of $$F = \frac{3}{2} \to F^{^{\prime}} = \frac{5}{2} \to F^{^{\prime\prime}} = \frac{5}{2}$$ hyperfine transition of ^223^Ra for the second excitation transition with reference to ^224^Ra (*), ^226^Ra (#) and ^228^Ra ($) are also marked. (**A**) Bandwidth of both excitation lasers is 0 MHz; Peak power density of the excitation and ionization lasers are 10 W/cm^2^, 10 W/cm^2^ and 10 W/cm^2^ respectively; Full angle divergence of the atomic beam is 0° (i.e., No Doppler broadening) (**B**) Bandwidth of both excitation lasers is 500 MHz; Peak power density of the excitation and ionization lasers are 10 W/cm^2^, 10 W/cm^2^ and 10 W/cm^2^ respectively; Full angle divergence of the atomic beam is 0° (i.e., No Doppler broadening) (**C**) Bandwidth of both excitation lasers is 0 MHz; Peak power density of the excitation and ionization lasers are 10 W/cm^2^, 10 W/cm^2^ and 10,000 W/cm^2^ respectively; Full angle divergence of the atomic beam is 0°. (i.e., No Doppler broadening) (**D**) Bandwidth of both excitation lasers is 0 MHz; Peak power density of the excitation and ionization lasers are 10 W/cm^2^, 10 W/cm^2^ and 10 W/cm^2^ respectively; Full angle divergence of the atomic beam is 30° (**E**) Bandwidth of both excitation lasers is 500 MHz; Peak power density of the excitation and ionization lasers are 10 W/cm^2^, 10 W/cm^2^ and 10,000 W/cm^2^ respectively; Full angle divergence of the atomic beam is 30°.

Two-dimensional contour of ionization efficiency has been calculated for the even isotope of Radium and plotted in Fig. 4. Expected resonance frequency position range of $$F^{^{\prime}} = \frac{5}{2} \to F^{^{\prime\prime}} = \frac{5}{2}$$ hyperfine transition of ^223^Ra isotope for the second excitation transition with reference to ^224^Ra(*),^226^Ra(#) and ^226^Ra($) isotopes has been shown in Fig. 4. In this graph, the variation in the ionization efficiency of even isotopes of Radium in the expected frequency range of $$F^{^{\prime}} = \frac{5}{2} \to F^{^{\prime\prime}} = \frac{5}{2}$$ hyperfine transition of ^223^Ra (of the second excitation transition) can be observed. Figure 4A has been generated by setting the laser bandwidth of 0 MHz (monochromatic laser), peak power density of 10, 10, 10 W/cm2 for the excitation and ionization lasers under Doppler free excitation. It can be seen from Fig. 4A that the ionization efficiency of non target isotopes varies by about three (^224^Ra) to four (^226^Ra, ^228^Ra) orders of magnitude within the expected resonance frequency range of $$F^{^{\prime}} = \frac{5}{2} \to F^{^{\prime\prime}} = \frac{5}{2}$$ hyperfine transition of ^223^Ra. Figure 4B is calculated under the same conditions as Fig. 4A except that laser bandwidth is set to 500 MHz. In this case, as a result of bandwidth of the excitation lasers, ionization efficiency contour is broadened. Within the expected resonance frequency range of $$F^{^{\prime}} = \frac{5}{2} \to F^{^{\prime\prime}} = \frac{5}{2}$$ hyperfine pathway, the ionization efficiency of ^224^Ra does not alter significantly while the ionization efficiency of ^226^Ra and ^228^Ra vary by three and four orders magnitude respectively. Figure 4C shows the ionization efficiency contour of even isotope calculated with similar conditions of Fig. 4A, except that in this case the power of the ionization laser has been raised to 10,000W/cm^2^. Due to the high power of the ionization laser, the ionization efficiency of non-target even isotope increases by three orders of magnitude at the resonance (0 MHz, 0 MHz), while its ionization efficiency within the expected $$F^{^{\prime}} = \frac{5}{2} \to F^{^{\prime\prime}} = \frac{5}{2}$$ hyperfine transition frequency range of the second transition of ^223^Ra varies by one (^224^Ra) to four (^226^Ra and ^228^Ra) orders of magnitude. Therefore, it can be concluded that under these conditions the saturation broadening has little influence on the degree of enrichment of the laser isotope process. Figure 4D shows the ionization efficiency of even isotope under similar conditions of Fig. 4A except that the Doppler broadening of the atomic ensemble has been considered for the present calculation. Even in this case, the ionization efficiency of even isotopes varies by two (^224^Ra), three (^226^Ra) and four (^228^Ra) orders of magnitude. Figure 4E shows the ionization efficiency contour of even isotope inclusive all the system parameters such as bandwidth (500 MHz), high power (10, 10, 10,000 W/cm^2^) and Doppler broadening (angular divergence of 30°). From Fig. 4E, the ionization efficiency of even isotopes varies by one (^224^Ra) to three (^226^Ra, ^228^Ra) orders of magnitude. Under these conditions, when the isotope shift of even isotopes of second excitation transition has been considered to be 0 MHz, the degree of enrichment of ^223^Ra is found to be ~ 20%. As mentioned earlier, the isotope shift of the second excitation can be about 50% of the first excitation transition; under this case the degree of enrichment of ^223^Ra isotope can reach a value of 93.6%. However, further experiments for the measurement of isotope shifts of the second excitation transition are necessary to exploit the photoionization scheme for laser isotope separation of ^223^Ra effectively.

### Radionuclidic purity of the enriched isotope

The utility of the enriched radionuclide for cancer therapy depends on it’s radionuclidic purity (must be differentiated from isotopic purity) in the enriched mixture. No-carrier added specific activity of a radioisotope can be calculated from its half-life using the following expression

11 $$S= \frac{4.174\times {10}^{23}}{{AMU\times T}_{1/2}}$$

where S is the specific activity (Bq/gm), AMU is the atomic weight of the isotope (amu) and T_1/2_ is the half-life of the isotope (Sec).

Radionuclidic purity (R_i_) of an isotope “i” can be calculated using the expression

12 $${R}_{i} (\%)= \frac{{S}_{i}*{f}_{i}}{\sum_{i}^{all}{S}_{i}*{f}_{i}}\times 100$$

where *S*_*i*_ is the specific activity of the isotope “i” (Bq/gm), *f*_*i*_ is the fractional abundance of the same isotope in the isotope mixture.

From the abundance and the no-carrier added specific activity of the isotopes in the enriched isotope mixture, the radionuclidic purity of ^223^Ra has been calculated to be 98.5%. The radionuclidic purity is adequate for the medical applications intended.

As mentioned earlier, considering that the isotope shift of the second excitation transition is 50% of the first excitation transition, the degree of enrichment can reach a value of 93.6%. In such a case the radionuclidic purity of the isotope mixture is calculated to be 99.9%.

## Conclusions

A three-step photoionization scheme has been theoretically investigated for the laser isotope separation of ^223^Ra. The effect of bandwidth, peak power density of the excitation and ionization lasers, Doppler broadening of the atomic ensemble, number density of the atoms on the laser isotope separation process has been studied. The system parameters for the optimum separation of ^223^Ra through this photoionization scheme have been derived. The effect of unknown parameters on the degree of enrichment has also been discussed. It has been theoretically shown that it is possible to produce ~ 20% enriched ^223^Ra isotope at a rate of 0.74 μg/h corresponding to the production rate of 435 patient doses per hour. The radionuclidic purity of ^223^Ra has been found to be 98.5% which is adequate for the intended application. This is the first ever study on the laser isotope separation of Radium isotopes.

## Data Availability

The datasets used and/or analysed during the current study available from the corresponding author on reasonable request.

## References

[1] Radchenko V, Morgenstern A, Jalilian AR, Ramogida CF, Cutler C, Duchemin C, Hoehr C, Haddad F, Bruchertseifer F, Gausemel H, Yang H, Alberto Osso J, Washiyama K, Czerwinski K, Leufgen K, Pruszynski M, Valzdorf O, Causey P, Schaffer P, Perron R, Maxim S, Wilbur DS, Stora T, Li Y (2021). Production and supply of α-particle–emitting radionuclides for targeted α-therapy. J. Nucl. Med..

[2] Delgado Bolton RC, Giammarile F (2018). Bone radionuclide therapy and increased survival with radium-223 is the way to go for nuclear medicine: The offer that oncologists cannot refuse. Eur. J. Nucl. Med. Mol. Imaging.

[3] Deshayes E, Roumiguie M, Thibault C, Beuzeboc P, Cachin F, Hennequin C, Huglo D, Rozet F, Kassab-Chahmi D, Rebillard X, Houédé N (2017). Radium 223 dichloride for prostate cancer treatment. Drug Des. Dev. Ther..

[4] Wang Le, Bin Lu, He M, Wang Y, Wang Z, Lingbin Du (2022). Prostate cancer incidence and mortality: Global status and temporal trends in 89 countries from 2000 to 2019. Front. Public Health.

[5] Kukleva E, Kozempel J, Vlk M, Micolova P, Vopalka D (2015). Preparation of 227Ac/223Ra by neutron irradiation of 226Ra. J. Radioanal. Nucl. Chem..

[6] Shishkin DN, Krupitskii SV, Kuznetsov SA (2011). Extraction generator of 223Ra for nuclear medicine. Radiochemistry.

[7] Pandit-Taskar N, Larson SM, Carrasquillo JA (2014). Bone-seeking radiopharmaceuticals for treatment of osseous metastases, part 1: A therapy with 223Ra-Dichloride. J. Nucl. Med..

[8] XOFIGO, Product Monograph, 2022, Bayer Inc., Canada.

[9] Dammalapati U, Jungmann K, Willmann L (2016). Compilation of spectroscopic data of radium (Ra I and Ra II). J. Phys. Chem. Ref. Data.

[10] Wendt K, Ahmad SA, Klempt W, Neugart R, Otten EW, Stroke HH (1987). On the hyperfine structure and isotope shift of radium. Zeitschrift für Physik D Atoms Mol. Clust..

[11] Raeder S, Lassen J, Heggen H, Teigelhofer A (2014). In-source spectroscopy on astatine and radium for resonant laser ionization. Hyperfine Interact..

[12] Day Goodacre T, Billowes J, Binnersley CL, Bissell ML, Chrysalidis K, Cocolios TE, de Groote RP, Farooq-Smith GJ, Fedorove DV, Fedosseev VN, Flanagan KT, Franchoo S, Garcia Ruiz RF, Gins W, Heinke R, Koszorús Á, Lynch KM, Marsh BA, Molkanov PL, Naubereit P, Neyens G, Ricketts CM, Rothe S, Seiffert C, Seliverstov MD, Stroke HH, Studer D, Vernon AR, Wilkins SG, Wendt KDA, Yang XF (2018). Radium ionization scheme development: The first observed autoionizing states and optical pumping effects in the hot cavity environment. Spectrochim. Acta.

[13] Lynch KM, Wilkins SG, Billowes J, Binnersley CL, Bissell ML, Chrysalidis K, Cocolios TE, Day Goodacre T, de Groote RP, Farooq-Smith GJ, Fedorov DV, Fedosseev VN, Flanagan KT, Franchoo S, Garcia Ruiz RF, Gins W, Heinke R, Koszorús Á, Marsh BA, Molkanov PL, Naubereit P, Neyens G, Ricketts CM, Rothe S, Seiffert C, Seliverstov MD, Stroke HH, Studer D, Vernon AR, Wendt KDA, Yang XF (2018). Laser-spectroscopy studies of the nuclear structure of neutron-rich radium. Phys. Rev..

[14] Hassouneh O, Salah W (2022). Relativistic calculations of the Landé gJ-factors, and hyperfine parameters in Ra-223 isotope. Eur. Phys. J. Plus.

[15] Shore BW (1990). The Theory of Coherent Atomic Excitation: Simple Atoms and Fields.

[16] Suryanarayana MV (2021). Isotope selective three-step photoionization of ^176^Lu. J. Opt. Soc. Am..

[17] Lambropoulos P, Lyras A (1989). Theory of resonant ionization by broad-band radiation in the determination of isotopic abundances. Phys. Rev..

[18] Lyras A, Zorman B, Lambropoulos P (1990). Theory of doubly resonant ionization by broad-band radiation applied to the determination of isotopic abundances. Phys. Rev..

[19] Bushaw BA, Nörtershäuser W, Wendt K (1999). Lineshapes and optical selectivity in high-resolution double-resonance ionization mass spectrometry. Spectrochim. Acta.

[20] Sakabe S, Izawa Y (1992). Simple formula for the cross sections of resonant charge transfer between atoms and their positive ions at low impact velocity. Phys. Rev..

[21] Bergmann E, Bopp P, Dorsch CH, Kowalski J, Träger F, ZuPutlitz G (1980). Nuclear charge distribution of eight Ca-nuclei by laser spectroscopy. Zeitschrift für Physik Atoms Nuclei.

[22] Pery A (1954). Isotope shifts in the atomic spectrum of calcium. Proc. Phys. Soc..

[23] Bushaw BA, Cannon BD (1997). Diode laser based resonance ionization mass spectrometric measurement of strontium-90. Spectrochim. Acta.

[24] King WH (1984). Isotope shifts in atomic spectra.

